# Non–Negligible Ecological Risks of Urban Wetlands Caused by Cd and Hg on the Qinghai–Tibet Plateau, China

**DOI:** 10.3390/toxics11080654

**Published:** 2023-07-28

**Authors:** Lei Wang, Xufeng Mao, Xiuhua Song, Xiaoyan Wei, Hongyan Yu, Shunbang Xie, Lele Zhang, Wenjia Tang

**Affiliations:** 1 Key Laboratory of Tibetan Plateau Land Surface Processes and Ecological Conservation, Ministry of Education, Qinghai Normal University, Xining 810008, China; 18234145683@163.com (L.W.); zhang1986lele@163.com (L.Z.); 2 Qinghai Province Key Laboratory of Physical Geography and Environmental Process, College of Geographical Science, Qinghai Normal University, Xining 810008, China; 3 Academy of Plateau Science and Sustainability, People’s Government of Qinghai Province and Beijing Normal University, Xining 810016, China; 4 Management and Service Center for Huangshui National Wetland Park, Xining 810016, China; songxh5087@163.com (X.S.); hnwpxie374@126.com (S.X.); 5 School of Economics and Management, Qinghai Normal University, Xining 810008, China; nanpishu1234@yeah.net; 6 Management and Service Center of Qilian Mountain National Park, Xining 810008, China; yuhy502@yeah.net; 7 State Key Laboratory for Environmental Protection Monitoring and Assessment of the Qinghai–Xining Plateau, Xining 810007, China; qhtsy@126.com

**Keywords:** urban wetlands, soil heavy metals, ecological risk, spatial variability, source identification

## Abstract

The Huangshui National Wetland Park (HNWP) is a unique national wetland park in a city on the Qinghai-Tibetan Plateau, containing three zones: Haihu, Beichuan, and Ninghu. In this study, a total of 54 soil samples (18 sampling points with depths of 0–10 cm, 10–20 cm, and 20–30 cm) were collected in these three zones, and the contents of heavy metals (Cr, Cd, Cu, Hg, Ni, Pb, Zn, and As) of each sample were determined. The ecological risk of eight kinds of heavy metals was evaluated by using the geo–accumulation index ($I_{geo}$), and the ecological risk–controlling effect of the Xining urban wetlands on heavy metals was explored by comparative analysis, and the possible sources of heavy metals in the soil were analyzed via correlation analysis and principal component analysis (PCA). The results revealed that the total heavy metal concentration order was Haihu > Beichuan > Ninghu zone. As and Cu presented vertical accumulation characteristics in the surface and lower horizon, respectively. Cr, Cd, Hg, Ni, Pb, and Zn accumulated downwards along the depth. On the spatial scale, the enrichments of Cd and Hg brought non-negligible ecological risks in plateau urban wetlands. The results of PCA indicated that soil heavy metals mainly came from compound sources of domestic and atmospheric influences, traffic pollution sources, and industrial pollution sources. The study has revealed that human activities have inevitable negative impacts on wetland ecosystems, while the HNWP provides a significant weakening effect on heavy metal pollution.

## 1. Introduction

With the rapid socio–economic development and urbanization, pollutants of anthropogenic origin gradually accumulate in the soil. Many countries face a common environmental problem, especially the increase in heavy metals in urban wetlands soils, which has attracted worldwide attention [1,2]. Heavy metals are difficult to degrade, highly toxic, and easily transported, endangering entire ecosystems and affecting human health through the food chain. Although some areas are remote or have no industrial activities, such as mangroves and grasslands, heavy metal accumulation has occurred, harming the environment and humans, mainly due to waterborne transport and long-range transport of contaminant mining [3,4,5], which indicates that there is a possibility of pollution risk in any ecosystem. Meanwhile, with increasing urbanization and frequent human activities, pollutants like heavy metals have accumulated in urban wetlands on the Qinghai–Tibet Plateau, where the ecosystem is fragile and sensitive, which is more worthy of our attention. However, limited research has been performed on the ecological risks of heavy metals in plateau urban wetlands. Therefore, studying the distribution characteristics, potential ecological risks, and pollution source analysis of heavy metals in urban wetland soils is essential for pollution prevention and environmental protection [6].

In recent decades, there have been many studies about heavy metal migration and accumulation, impact factors, and significant sources of wetlands soils at home and abroad [7,8]. On the one hand, the heavy metal concentrations in soil are impacted by natural factors, such as parent materials or rock–forming minerals, which tend to make the concentration of Cr and As in soil higher [9]; the volatile element, Hg, has accumulated in excess on the Tibetan Plateau due to monsoon effects [10,11]. On the other hand, soil heavy metals mainly originate from human activities, including iron and steel plants or chemical factories around cities, automobile exhaust in cities, agricultural fertilizers, and urban sewage discharge, etc. [12,13]. There are few reports on heavy metals in alpine urban wetland soils, comparative analysis with other urban wetlands or wetland soils, and the ecological risk of rapid urbanization on them.

In terms of heavy metal risk evaluation, most of the evaluation indexes such as bioconcentration factor (BFC), geo–accumulation index ($I_{geo}$), risk assessment index (RAC), toxicity ecological index (TRI), potential ecological risk index ($E_{r}^{i}$), or heavy metal morphology evaluation method (RSP) are used to quantify the degree of heavy metal enrichment, and ecological risk level in soil [14,15,16]. In the source analysis of heavy metals, studies have classified heavy metals that may have similar sources into the same group according to the spatial distribution of heavy metal concentrations, mainly through correlation analysis, principal component analysis, cluster analysis or multiple regression analysis, and other methods [17,18]. However, all of the above do not consider the influence of soil properties or environmental factors, while the distribution and morphology of soil heavy metals are related to soil pH, iron and manganese oxides, and other physicochemical properties [19,20,21]. Therefore, this study has used multiple methods to evaluate the heavy metal concentration of urban wetland soil to obtain more objective and accurate results.

The Huangshui National Wetland Park (abbreviated as HNWP, the same below) is located in the Qinghai–Tibet Plateau, known as the “World’s Third Pole” and “Asia’s Water Tower” [22,23]. HNWP is a typical plateau urban wetland in northwest China, is the “City Kidney” of Xining, and plays a critical role in protecting the ecological balance of highland cities and conserving urban water [24,25]. Due to the unique geographical features and climatic conditions of HNWP, its ecosystem is fragile and sensitive, and minor heavy metal contamination can significantly impact the entire urban ecosystem [26,27]. Previous studies have shown that heavy metals in urban wetland soils have accumulated somewhat. Some heavy metal concentrations have exceeded relevant national standards, and the problem of heavy metal pollution in urban wetlands has begun to emerge [28]. Therefore, the HNWP was selected in order to explore the ecological effects of urban wetlands on heavy metal risks.

In summary, this study aims to: (1) determine the concentrations of soil heavy metals (As, Cr, Cd, Cu, Hg, Ni, Pb, and Zn) in the urban wetlands of Xining and reveal the spatial distribution characteristics of heavy metals in soil; (2) compare HNWP with other urban wetlands to explore the enrichment of heavy metals in Xining urban wetlands, and evaluate the ecological risk; (3) identify which heavy metals have pose an ecological risk in each zone and analyze the primary sources of heavy metals in soil. The results can provide the scientific basis for soil pollution control and ecological environment protection in the plateau urban wetlands.

## 2. Materials and Methods

### 2.1. Site Description

HNWP (36°33′41″–36°44′42″ N, 101°37′06″–101°54′50″ E) is located in Xining, Qinghai Province. The total area of the wetland is 241.41 hm^2^, including three zones of Haihu, Beichuan, and Ninghu. These three zones all belong to urban wetlands of HNWP, but there are differences in spatial location and the extent of human activity. The Haihu and Beichuan zones are located upstream, while the Ninghu zone is located downstream of the Huangshui River. The water source of the wetlands mainly comes from the Huangshui River and the Beichuan River. Among the three zones, the Haihu zone has the most commercial activities and residential areas, while the Beichuan zone has slightly fewer, and the Ninghu zone is far from the urban center and has the least human activity. The topography of Xining is high in the northwest and low in the southeast; the landscape is dominated by shallow hills and river valley terraces, with an average elevation of 2261 m. The average annual temperature is 5.5 °C; the average annual sunshine is 2510.1 h; the temperature difference between day and night is significant, the solar radiation is intense, and it belongs to the continental plateau semi–arid climate. The average annual rainfall is about 500 mm, unevenly distributed within the year, with more than 80% of precipitation concentrated from June to September and evaporation of 1363.6 mm [29,30]. The HNWP runs through Xining from west to east, benefiting 40% of the province’s population, and has a well–developed transportation network and intensive traffic flow. Xining, where the HNWP is located, is in a basin with industrial parks to the south, aluminum plants to the north, and primary pollution sources such as steel plants to the west [31,32].

### 2.2. Soil Sampling

**Figure 1:**
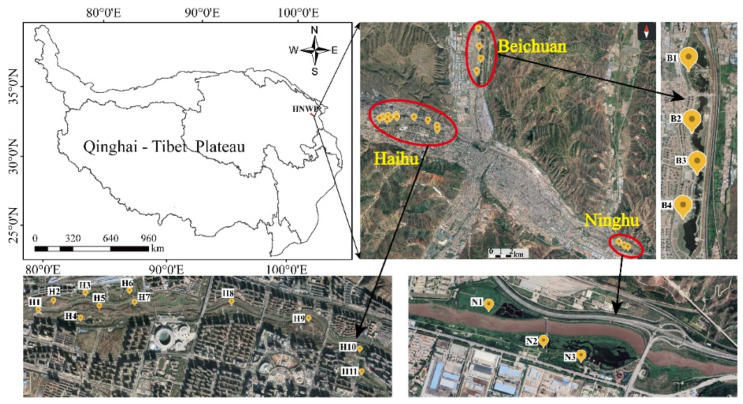
Location of the sampling sites in Huangshui National Wetland Park (HNWP).

Considering the actual area, road accessibility, uniform distribution spatially of sampling points, and vegetation diversity, a total of 18 soil sampling points were laid out in HNWP (Figure 1); spatially divided into three groups, including 11 samples in Haihu zone (H1–H11), 4 samples in Beichuan zone (B1–B3) and 3 samples in Ninghu zone (N1–N3); and each sample point was accurately located via Global Position System (GPS). At each sample point, soil was collected at three horizons: 0–10 cm, 10–20 cm, and 20–30 cm via auger boring, and a total of 54 soil samples were collected for testing. The samples were then placed in polyethylene plastic bags to prevent contamination and marked with a marker pen. The soil samples were dried with natural air, and gravels and roots were removed, as needed. The soil samples for analyzing chemical properties and heavy metals concentration went through 10, 60, and 100 mesh screens.

### 2.3. Chemical Analysis

Soil pH was measured via pH meter (soil:water = 1:2.5). Soil organic carbon was determined via titration with FeSO_4_ after digestion with K_2_Cr_2_O_7_–H_2_SO_4_ solution. Total nitrogen (TN) was measured via the semi–micro Kjeldahl method, and total phosphorus (TP) was analyzed using the Mo–Sb colorimetric method. For heavy metal analysis, soil samples were digested with a typically concentrated acid mixture (HNO_3_–HClO_4_–HF), and the concentrations of Cd, Cr, Cu, Ni, Pb, and Zn were determined via inductively coupled plasma–mass spectrometer (ICP–MS); samples were digested using Aqua Regia (HF, HClO_4_) and the contents of As and Hg were determined using an atomic fluorescence photometer (AFS–8130) [33,34]. The standard reference material of ESS–2 from China was employed for quality assurance and quality control, and the recovery rate of eight heavy metals ranged between 95% and 110%. The practical limits for the analysis of Cd, Cr, Cu, Ni, Pb, Zn, As, and Hg were 0.09, 2, 0.6, 1, 2, 1, 0.4, and 0.002 mg·kg^−1^, respectively. Three replicates were set for each metal, which were analyzed simultaneously.

### 2.4. Evaluation Methods

#### 2.4.1. Geo–Accumulation Index

The geo–accumulation index ($I_{geo}$), also known as the Muller index, is widely used in quantitatively evaluating heavy metal pollution. This method integrates the influence of geological background and human activities and can intuitively reflect the enrichment degree of exogenous heavy metals in soil [35]. The formula is:

(1) $$I_{geo}=\log_{2}\left(\frac{C_{i}}{KB_{i}}\right)$$

where $I_{geo}$ is the geo–accumulation index; *C_i_* is the measured content of element *i* in soil, mg·kg^−1^; *B_i_* is the background value of element *i*, mg·kg^−1^, which is the background value of soil in Qinghai Province in this study; *K* is the correction factor, which is used to correct for the variation of background value caused by geological factors, usually taken as 1.5. The grading criteria of $I_{geo}$ are shown in Table 1.

#### 2.4.2. Geo–Accumulation Index Potential Ecological Risk Assessment

Hakanson proposed the potential ecological risk index (RI) in 1980, which introduced the toxicity response coefficient of elements and was widely used in the evaluation of the degree of soil heavy metal pollution and ecological risk [6]. For determining whether heavy metals in soil pose pressure on the ecological environment of Xining urban wetlands, we selected the potential ecological risk factor ($E_{r}^{i}$) to evaluate the possible risk posed by heavy metals [28,36]. The formula is:

(2) $$E_{r}^{i}=T_{r}^{i}\times C_{r}^{i}=T_{r}^{i}\times \frac{C_{s}^{i}}{C_{n}^{i}}$$

(3) $$\mathrm{RI}=\sum_{i=1}^{n}E_{r}^{i}$$

where $E_{r}^{i}$ is the potential ecological risk factor of heavy metal element *i*; $C_{r}^{i}$ is the enrichment coefficient of element *i*; $C_{s}^{i}$ is the measured content of element *i*, mg·kg^−1^; $C_{n}^{i}$ is the reference value of element *i*, the study selected the environmental background value of Qinghai province, mg·kg^−1^; $T_{r}^{i}$ is the toxicity coefficient of element *i*. The toxicity coefficients of Hg, Cd, As Cu, Pb, Ni, Cr, and Zn were 40, 30, 10, 5, 5, 5, 2, and 1, respectively. RI represents the potential ecological risk index of heavy metals. The grading criteria for $E_{r}^{i}$ and RI are shown in Table 1.

### 2.5. Data Analysis

This study used Pearson correlation analysis to examine the correlation between soil pH, TOC, TN, TP, and heavy metal (Hg, Cd, As, Cu, Pb, Ni, Cr, Zn) content. A one-way analysis of variance (ANOVA) was used to determine the differences in the concentration of elements between the three zones. Principal component analysis (PCA) was applied to identify the primary sources of heavy metals. All data, statistics, and analyses were conducted with SPSS (Version 22.0, IBM Corporation, Armonk, New York, NY, USA), and all graphs and tables were created using Origin (Version 2022, OriginLab Corporation, Northampton, MA, USA) and Excel (Version 2019. Microsoft Corporation, Redmond, WA, USA).

## 3. Results

### 3.1. Concentrations of Heavy Metals in Soil

The heavy metals content of soils at different depths in HNWP are shown in Table 2. The highest concentrations of heavy metals in 0–10 cm and 20–30 cm soils were for Pb, with 13.97–793.12 and 18.27–737.52 g·kg^−1^, respectively. And the highest content of Zn in 10–20 cm soils was 35.03–417.27 g·kg^−1^. The lowest concentrations of heavy metals in soils at different depths was for Hg. The average concentration of heavy metals in soils at different depths was in the following order: Zn > Pb > Cr > Ni > Cu > As > Cd > Hg.

**Figure 2:**
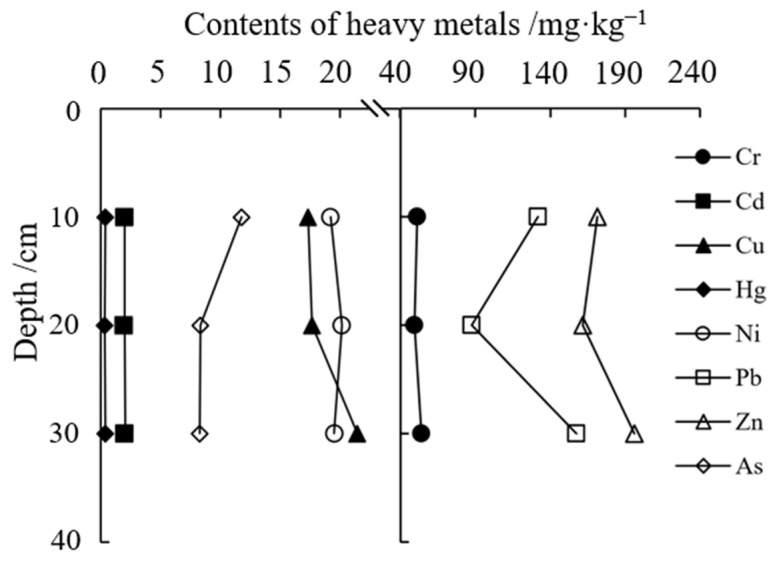
Distribution characteristics of soil heavy metals in soils with different depths in HNWP.

The accumulation and migration characteristics of heavy metals in soil profiles were shown in Figure 2. The content of As gradually decreased with increasing soil depth, and the average content in 20–30 cm soil decreased by 29.97% compared with 0–10 cm. The content of Cu increased along the downward profile, and the average content in 20–30 cm soil increased by 23.70% compared with 0–10 cm soil. The content of Cr, Cd, Hg, Pb, and Zn showed a decrease, and then an increase in the vertical direction of the profile, and their concentrations in 20–30 cm were higher than that in 0–10 cm and 10–20 cm. The Ni content was characterized by increasing and then decreasing vertically.

**Figure A1:**
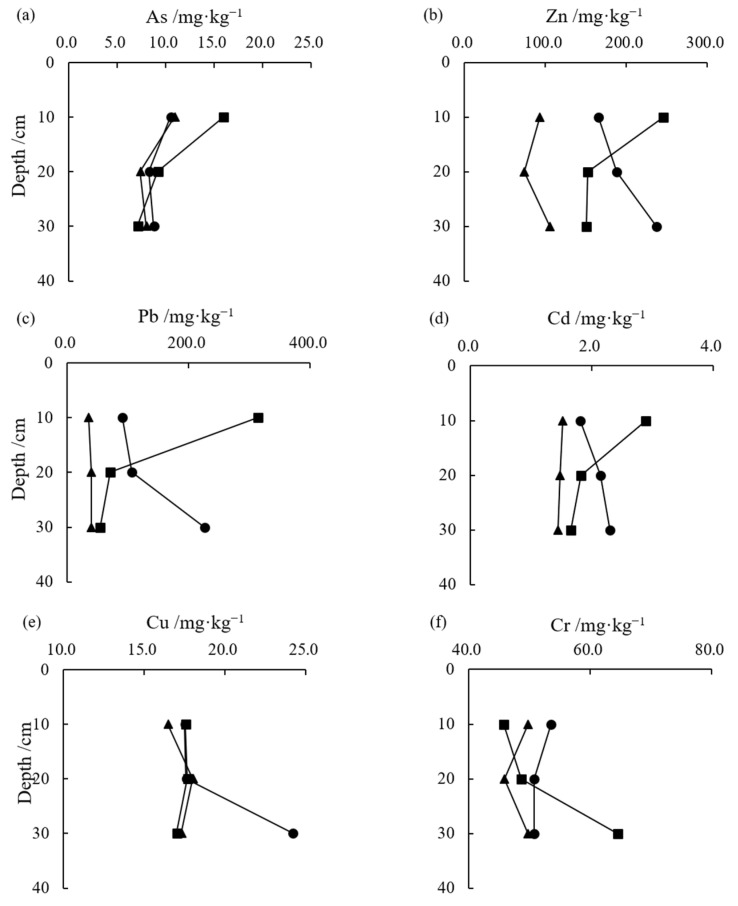

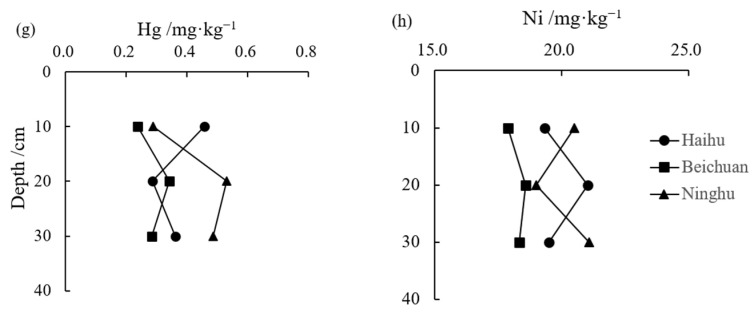
Distribution characteristics of heavy metals in soil profiles in different regions ((**a**–**h**), refer to heavy metal As, Zn, Pb, Cd, Cu, Cr, Hg, and Ni, respectively).

The vertical profile distribution of eight kinds of heavy metals in different zones of HNWP was shown in Figure A1. The As content demonstrated a surface clustering phenomenon in different zones, and Cu increased along the soil profile with prominent lower accumulation characteristics. Cr, Cd, Hg, Ni, Pb, and Zn concentrations showed different profile variation characteristics in different zones. However, the average concentrations in HNWP showed that the content in 20–30 cm soil was higher than that in 0–10 cm, indicating a tendency to accumulate in the lower horizon (Table 2).

### 3.2. Spatial Distribution Characteristics of Soil Heavy Metal Concentration

**Figure 3:**
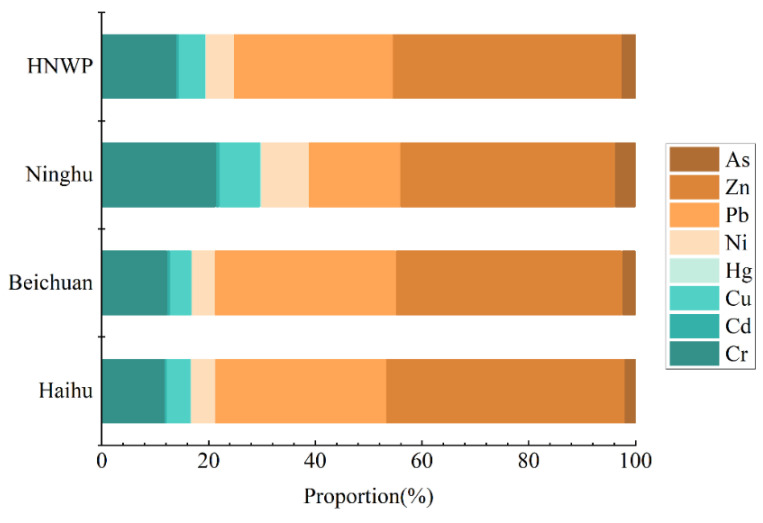
Concentrations of heavy metals in HNWP soil.

Figure 3 shows the percentage of concentrations for each heavy metal in HNWP. The highest and lowest concentrations were Zn (42.88%) and Hg (0.10%). The average concentrations of Cr, Cd, Cu, Hg, Ni, Pb, Zn, and As were 51.05, 1.90, 18.14, 0.36, 19.47, 108.99, 157.23, and 9.56 g·kg^−1^, respectively. The concentration of Zn in the Haihu, Beichuan, and Ninghu zones was significantly higher than other heavy metals. The total amount of eight different heavy metals in the Haihu zone was 442.23 g·kg^−1^, and in the Beichuan and Ninghu zones, it was 431.51 and 226.41 g·kg^−1^, respectively. The total concentrations of heavy metal in soil from highest to lowest spatially was: Haihu > Beichuan > Ninghu zone.

**Figure 4:**
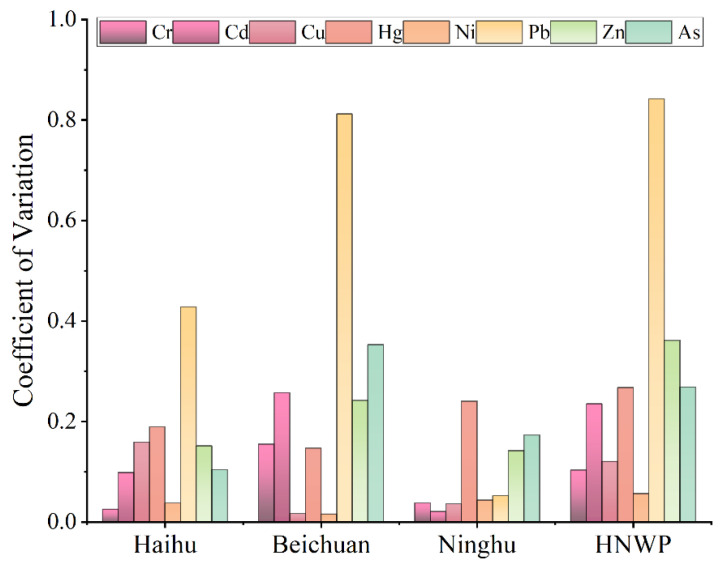
Coefficients of variation of heavy metal content in HNWP soil.

The degree of spatial variability of heavy metal concentrations can be characterized with the coefficient of variation (CV). The spatial variation of Pb content in the Haihu zone was the largest, with a CV of 0.43 (Figure 4), which is presumed to be influenced by human activities and may exist point source pollution; the CV of Hg was the second highest (0.19), which has a higher possibility of surface source pollution. The spatial variation of Cr, Cd, Cu, Ni, Zn, and As concentration was moderate, and their CVs were only 0.03–0.16, which was a weak variation, indicating that the possibility of surface pollution was slight. Similar to the Haihu zone, the variation of Pb concentration in the Beichuan zone fluctuated considerably, and its CV value reached a high value (0.81). The concentrations of various heavy metals in the Ninghu zone were more uniform overall, with slight fluctuation. However, the CV values of Hg were higher than other heavy metals, and Hg concentrations in this zone exceeded those in Haihu and Beichuan (Figure 2), indicating that point source pollution of Hg may exist in Ninghu.

### 3.3. Accumulation and Ecological Risks of Soil Heavy Metals

**Figure 5:**
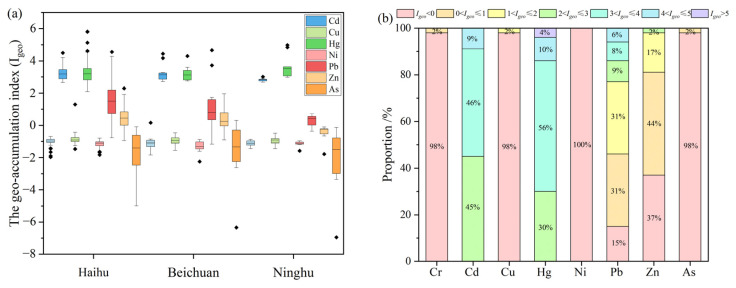
Geo–cumulation index ($I_{geo}$) of the heavy metals ((**a**) refers to the box plot of $I_{geo}$, (**b**) refers to the proportion of heavy metals with different $I_{geo}$ levels).

Figure 5 shows the $I_{geo}$ values of heavy metals in HNWP. The $I_{geo}$ values from highest to lowest were: Hg (3.58), Cd (3.18), Pb (1.80), Zn (0.38), As (−1.14), Cu (−0.88), Cr (−1.04) and Ni (−1.19). The $I_{geo}$ values of Cr, Cu, Ni, and As were all negative, indicating that the four heavy metals belonged to the uncontaminated level (Figure 5a,b). At the same time, Pb and Zn were moderately enriched and slightly enriched, and Hg and Cd were heavily enriched. the $I_{geo}$ values of Cd, Pb, and Zn varied among the different zones, with the $I_{geo}$ values of the Ninghu zone being lower than those of the Haihu and Beichuan zones.

**Figure 6:**
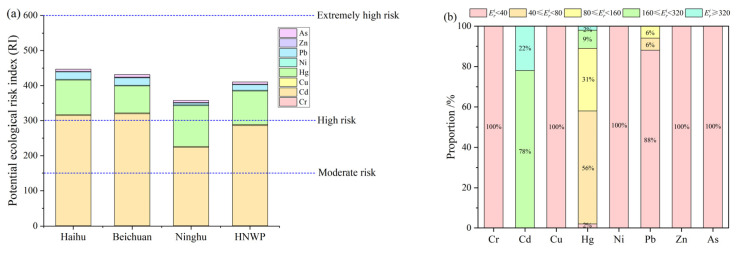
Potential ecological risk index ($E_{r}^{i}$) of soil heavy metals ((**a**) refers to cumulative bar graph of $E_{r}^{i}$ of heavy metals in soil in different regions, (**b**) refers to the proportion of heavy metals with different $E_{r}^{i}$ levels).

Figure 6 shows the $E_{r}^{i}$ values of heavy metals and their graded ratios in each zone of Xining urban wetlands. The $E_{r}^{i}$ values of the heavy metals from highest to lowest were Cd > Hg > Pb > As > Cu > Ni > Zn > Cr. The $E_{r}^{i}$ values of Cd and Hg were larger and posed a higher risk of harm to the environment, while the $E_{r}^{i}$ values of Cr, Cu, Ni, Pb, Zn, and As were <40, all of which belonged to the slight risk level. The potential ecological risk index (RI) values of Haihu, Beichuan, and Ninghu zones were 446.85, 431.31, and 357.55, respectively, and the potential ecological index of HNWP was 410.68. The RI of HNWP belonged to the substantial risk level, but if we exclude Cd and Hg, which have the highest $E_{r}^{i}$ values, the RIs of the remaining six heavy metals were below 150, and the potential ecological risk of heavy metals was slight at this time.

## 4. Discussion

### 4.1. Regulation for Ecological Risks of Soil Heavy Metals in HNWP

The presence of heavy metals in surface soils is an essential indicator for analyzing environmental conditions. According to the background values of soils in Qinghai Province and the national soil environmental quality level I standard, the concentrations of Cr, Cu, Ni, and As in 0–10 cm were within the acceptable range [37], but Cd, Pb, Hg, and Zn exceeded it to varying degrees [38,39]. Cd, Hg, and Pb in particular were found to be strongly enriched (Table 2), which means that Xining urban wetlands have an obvious purification function for Cr, Cu, Ni, and As. Meanwhile, Xining urban wetlands have also become a “sink” for Cd, Pd, Hg, and Zn pollution. Furthermore, accumulating heavy metals here has curbed their spread to the surrounding urban areas and reduced the ecological risk of heavy metals on the environment.

There were differences in the concentration of heavy metals in the three zones of HNWP, and the concentration of heavy metals in the Ninghu zone was significantly lower. On the one hand, because the Haihu zone is located in the main urban area of Xining City, it receives more daily discharge of domestic wastewater, resulting in a higher concentration of heavy metals in the soil. With the increase in urbanization in Xining City, the development of commercial buildings and residential areas near Haihu and Beichuan zones is more intense, and the probability of heavy metals entering soil and accumulating during production and daily life is higher. On the other hand, the Ninghu zone is downstream of the Xining section of the Huangshui River, and most of the sediments containing heavy metals are deposited upstream. Although the Ninghu zone is less affected by human activities and the ecological environment can be better protected, the ecological risk is gradually increasing due to the development and utilization of the surrounding land. In summary, the pressure of HNWP on risk regulation is gradually increasing. However, the ecological risk of the Ninghu zone is negligible at this stage, and the wetlands purification potential is remarkable.

### 4.2. Comparison between HNWP and Other Wetlands in China

The study compared the concentrations of heavy metals in HNWP soils with other urban wetlands in China. The results showed that the concentration of Pb in HNWP was higher than other wetlands and the average heavy metals concentrations of Chinese wetlands, and the ecological risk in HNWP was the strongest. In contrast, the Pb content of Zalong wetland in Heilongjiang was low, but its As content exceeded that of HNWP. In addition, similar to the Caohai wetland in Guizhou, the concentrations of Hg and Zn in HNWP were also higher, and the pollution level was also higher. Previous studies have found that the leading causes of Hg and Zn pollution in the Caohai wetlands were industrial wastewater (electroplating, metal products, and textiles) and untreated domestic water [40,41], similar to the pollution sources in the current study. The low Cr concentration in HNWP and the Zalong wetland may be related to soil properties [3,42]. The Zalong wetland is a medium–temperate continental monsoon climate with an average annual temperature of 3.5 °C, and the soil type is mainly black calcium soil with high organic matter content [43]. The HNWP has high altitude and intense solar radiation. However, there is a substantial temperature difference between day and night, temperatures are low in winter, and the soil type is mainly chestnut calcium soil with strong alkalinity [23]. Wang et al. found that soil organic carbon content and soil acidity and alkalinity affect Cr concentration in soil and its associated toxicity [15]. In this study, the pH of HNWP surface soil was found to be 8.3, and the organic carbon content was 9.46 g·kg^−1^, the main reason for the low Cr concentration. The Cu and Ni content in HNWP was low compared to other regions, and the background values of soil in Qinghai province were also low for these two elements, so Cu and Ni concentrations are related to the particular geological lithological background of Qinghai Province.

The heavy metal concentrations in HNWP soils were significantly different from the average concentration of heavy metals in Chinese wetlands [16, 43,44,45,46,47,48,49,50]. The concentrations of Cr, Cd, Cu, Ni, and As were significantly lower than the average value of Chinese wetlands, and the Zn content was similar. However, the Hg concentration was significantly higher (Table 3), which reflected that Hg had accumulated substantially in HNWP. The plateau urban wetlands are characterized by very cold temperatures and low oxygen levels. The unique climatic characteristics make soil and vegetation differ significantly from other urban wetlands, and thus, the ecosystem is more fragile. If no measures are taken to improve the situation of high Hg content, the heavy metal purification function of HNWP will be significantly affected.

### 4.3. Influence Factors and Pollution Sources of Heavy Metals in Soils

**Figure 7:**
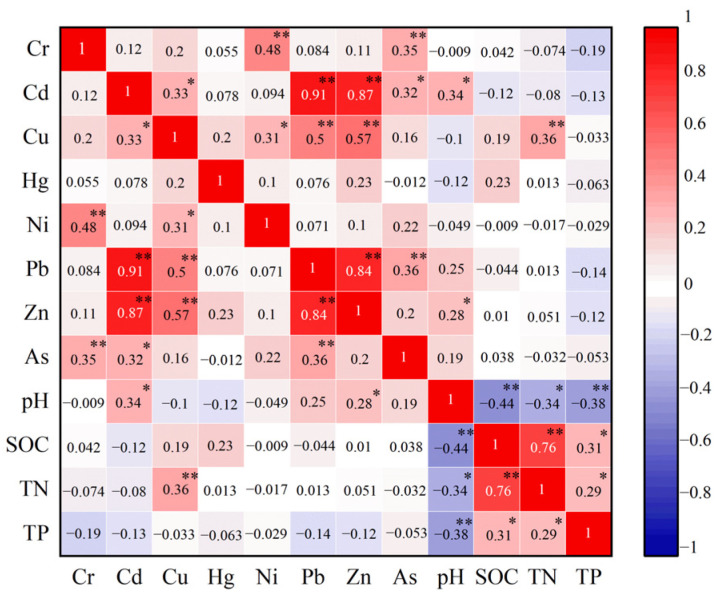
Correlation analysis between concentrations of heavy metals in soil and soil chemical properties. The numbers marked in the figure represent the correlation coefficients. Red represents a positive correlation, while blue represents a negative correlation; the darker the color, the stronger the correlation. ** indicates an extremely significant correlation (*p* < 0.01), and * indicates a significant correlation (*p* < 0.05).

The correlation coefficient between heavy metals and soil properties (Figure 7) indicated that there was a weak positive correlation between soil pH and Cd, as well as between soil pH and Zn (correlation coefficients were 0.34 and 0.28, respectively, *p* < 0.05) because the soil has a remarkable ability to remove heavy metals as its pH increases. It has been shown that dissolved heavy metals can exhibit physicochemical effects such as adsorption and sedimentation with the increase of soil alkalinity, thereby causing an increase in heavy metal concentration [15,51]. There was an extremely significant positive correlation between total nitrogen and Cu (correlation coefficient was 0.36, *p* < 0.01) because heavy metals such as Cu have a solid organic binding capacity, and the activity of heavy metal ions decreases with increasing nitrogenous organic matter [42], leading to an increase in heavy metal levels. From this, it can be seen that the soil environment significantly influences the accumulation of heavy metals. Correlation analysis can identify homologous heavy metals, since heavy metals of the same origin are correlated in terms of their concentration [17]. Cr, Cu, Pb, and Zn, as well as Cr and Ni in this study, are significantly correlated and may have the same source, while heavy metals with insignificant correlations may have multiple sources.

**Figure 8:**
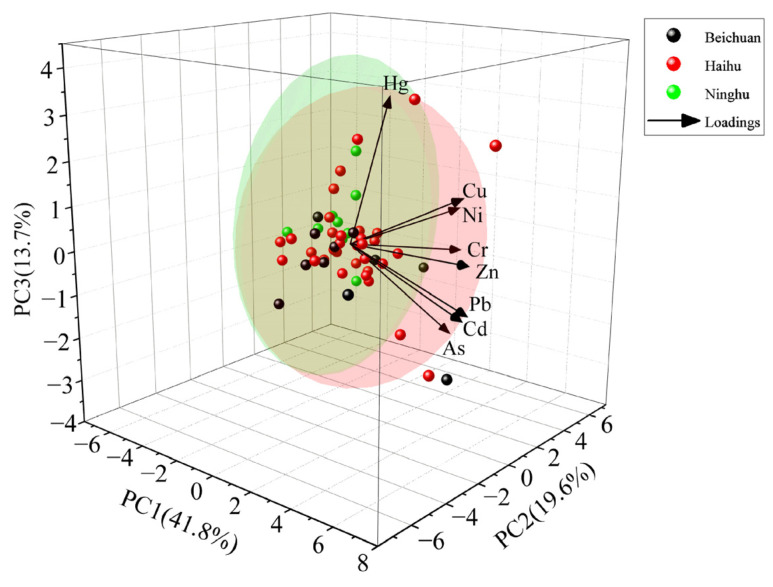
Factor load for principle component analysis.

Due to the rapid development of urban life, transportation, and tourism in Xining, the influence of human activities on the enrichment of heavy metals in HNWP has become more vital than soil factors such as pH and total nitrogen, which makes heavy metal pollution complex and multisource [21]. The principal component analysis (PCA) method can identify the primary sources of heavy metal contamination. There were three principal components (PC1, PC2, and PC3 with a cumulative contribution of 80.0%) identified with PCA, which can explain most of the heavy metal sources. The Kaiser–Meyer–Olkin (KMO) value was 0.806, and the significance coefficient of Bartlett’s test was less than 0.01. As can be seen from the PCA plot (Figure 8), Pb, Zn, Cd, and Cu have high loadings on PC1, and combined with the significant correlation between both Pb–Zn–Cd–Cu, it can be assumed that the sources of these four heavy metals were consistent. Motor vehicle metal parts, vehicle exhaust, and mechanical lubricants were the primary sources of Zn, Cd, and Cu, and Pb was the marker element of motor vehicle pollution [40]. Due to the proximity of Xining urban wetlands to roads and the heavy traffic, the wear and tear of parts and exhaust emissions can easily lead to the deposition of heavy metals in the soil. Therefore, PC1 can represent the source of traffic pollution.

The high loadings of Cr and Ni on PC2 and the highly significant correlation between Cr–Ni reflect a common source for them. It has been shown that Cr and Ni enrichment was associated with manufacturing and battery related industries, and industrial activities were significant causes of heavy metals accumulation in soil [24,52]. Therefore, PC2 can represent an industrial source of pollution.

As and Hg have high loadings on PC3 but are not significantly correlated, suggesting that As and Hg may have different sources. The possible sources of As were, on the one hand, the presence of urban rainwater, domestic sewage inflow, and industrial wastewater discharge in HNWP, and on the other hand, fertilizers and animal manure applied due to the planting of flowers and trees. Hg is associated with atmospheric deposition [10,20], and atmospheric deposition after long distance transport under the influence of monsoons may be the primary source of Hg in Xining urban wetlands. It has been reported in the past that Hg is volatile, India and China were the countries with the highest Hg emissions in the world, and Hg could be transported to the Qinghai–Tibet Plateau due to wind, which will quickly cause excessive accumulation of Hg in the long run [25,53,54]. Therefore, PC3 reflects the combined effect of domestic waste and atmospheric activities on As and Hg and can be called a compound source of domestic pollution and atmospheric impact.

From the above, it can be seen that the eight kinds of heavy metals in HNWP in this study came from three primary sources: traffic, industry, urban living, and atmospheric impacts, consistent with the results of many previous studies. For example, Xu et al. attributed the accumulation of Cd and Pb to industrial sources through their study [39]; Jiang et al. found that Zn and Pb came from traffic emissions, Cr and Ni were closely related to industrial production, and As originated from agricultural and living wastewater when analyzing the sources of heavy metal pollution [55].

## 5. Conclusions

This study analyzed the concentration of heavy metals in soil vertically and horizontally in plateau urban wetlands, revealed the spatial distribution and enrichment characteristics of heavy metals in soil, evaluated their ecological risk levels, and explored the sources of heavy metal soil pollution. The main conclusions are as follows:

(1) The vertical distribution of As and Cu was characterized by clustering in the surface layer and accumulation in the lower layer, respectively. At the same time, Cd, Hg, Pb, Ni, Zn, and Cr showed an accumulation trend to the lower layer. On the spatial scale, the order of heavy metal content was Haihu > Beichuan > Ninghu zone.
(2) Both the whole HNWP and the three zones have reached a strong risk level for heavy metal pollution, with the main pollution contribution coming from Cd and Hg, but dropping to a slight risk level after excluding two heavy metal risks.
(3) In this study, HNWP has a significant weakening effect on heavy metal pollution. The primary sources of eight heavy metals include compound sources of living and atmospheric impact, traffic, and industrial pollution.

This study can provide a scientific basis for soil pollution control and ecological protection in plateau urban wetlands.

## Figures and Tables

**Table 1:** Classification standard of evaluation methods.

$I_{geo}$		$E_{r}^{i}$		RI	
Value	Category	Value	Class	Value	Class
$I_{geo}$ < 0	Uncontaminated	$E_{r}^{i}$ < 40	Slight risk	RI < 150	Slight risk
0 < $I_{geo}$ ≤ 1	Slight enrichment	40 ≤ $E_{r}^{i}$ < 80	Medium risk	150 ≤ RI < 300	Medium risk
1 < $I_{geo}$ ≤ 2	Moderately enrichment	80 ≤ $E_{r}^{i}$ < 160	Strong risk	300 ≤ RI < 600	Strong risk
2 < $I_{geo}$ ≤ 3	Moderately to heavily enrichment	160 ≤ $E_{r}^{i}$ < 320	Very strong risk	RI ≥ 600	Very strong risk
3 < $I_{geo}$ ≤ 4	Heavily enrichment	$E_{r}^{i}$ ≥ 320	Extremely strong risk		
4 < $I_{geo}$ ≤ 5	Heavily to extremely enrichment				
$I_{geo}$ > 5	Extremely enrichment				

**Table 2:** Concentrations of soil heavy metals in different soil depths in HNWP.

Depth (cm)	Heavy Metals (mg·kg^−1^)	Cr	Cd	Cu	Hg	Ni	Pb	Zn	As
0–10	Max	63.23	4.56	24.13	1.68	23.99	793.12	469.08	25.97
	Min	29.45	1.37	11.38	0.16	9.35	13.97	62.18	2.05
	Mean	51.21	2.00	17.34	0.38	19.20	131.84	171.70	11.78
	STD	8.74	0.85	2.80	0.34	3.50	188.25	114.96	6.41
10–20	Max	64.99	3.85	23.64	0.95	25.55	330.07	417.27	18.96
	Min	26.86	1.34	12.13	0.13	14.48	25.04	35.03	0.66
	Mean	49.45	1.97	17.67	0.34	20.15	87.45	161.60	8.33
	STD	10.38	0.66	2.91	0.19	3.41	71.75	86.36	4.83
20–30	Max	117.52	4.74	81.64	1.05	23.86	737.52	590.57	19.66
	Min	33.44	1.32	13.93	0.17	13.85	18.27	66.44	0.17
	Mean	53.68	2.02	21.45	0.37	19.51	157.54	196.40	8.25
	STD	16.69	0.83	14.90	0.24	2.81	194.90	128.79	6.10

**Table 3:** Comparison of heavy metal concentrations (mg·kg^−1^) in soils of present research and other wetlands.

Wetland	Province	Cr	Cd	Cu	Hg	Ni	Pb	Zn	As
Zhalong	Heilongjiang	46.47	0.155	18.17	0.065	–	21.38	52.09	10.26
Poyang Lake	Jiangxi	105.77	0.42	12.25	–	30.47	27.81	79.45	6.39
Dongting Lake	Hunan	91.33	4.39	36.27	0.19	46.36	54.82	–	25.67
Huixian	Guangxi	114.67	0.67	37.12	0.20	40.16	45.22	125.43	17.62
Caohai	Guizhou	116.4	3.2	24.1	0.61	54.9	49.8	171.0	–
Yilong Lake	Yunnan	86.73	0.76	31.40	–	35.99	53.19	86.82	15.46
HNWP	Qinghai	51.21	2.00	17.34	0.38	19.20	131.84	171.70	11.78
Mean of Chinese Wetlands	–	100.11	4.56	28.96	0.13	38.79	46.37	163.98	14.52
Background value of Qinghai	–	70.1	0.14	22.2	0.02	29.6	20.9	80.3	14.0
National Class I standard soil environmental quality	–	90.0	0.2	35.0	0.15	40.0	35.0	100.0	15.0

## Data Availability

Not applicable.
